# Teaching clinical handover with ISBAR

**DOI:** 10.1186/s12909-020-02285-0

**Published:** 2020-12-03

**Authors:** Annette Burgess, Christie van Diggele, Chris Roberts, Craig Mellis

**Affiliations:** 1 The University of Sydney, Faculty of Medicine and Health, Sydney Medical School - Education Office, The University of Sydney, Edward Ford Building A27, Sydney, NSW 2006 Australia; 2 The University of Sydney, Faculty of Medicine and Health, Sydney Health Professional Education Research Network, The University of Sydney, Sydney, Australia; 3 The University of Sydney, Faculty of Medicine and Health, The University of Sydney, Sydney, Australia; 4 The University of Sydney, Faculty of Medicine and Health, Sydney Medical School – Central, The University of Sydney, Sydney, Australia

**Keywords:** Clinical handover, ISBAR (Introduction, Situation, Background, Assessment, Recommendation), Patient safety, Interprofessional

## Abstract

Clinical handover is one of the most critical steps in a patient’s journey and is a core skill that needs to be taught to health professional students and junior clinicians. Performed well, clinical handover should ensure that lapses in continuity of patient care, errors and harm are reduced in the hospital or community setting. Handover, however, is often poorly performed, with critical detail being omitted and irrelevant detail included. Evidence suggests that the use of a structured, standardised framework for handover, such as ISBAR, improves patient outcomes. The ISBAR (Introduction, Situation, Background, Assessment, Recommendation) framework, endorsed by the World Health Organisation, provides a standardised approach to communication which can be used in any situation. In the complex clinical environment of healthcare today, ISBAR is suited to a wide range of clinical contexts, and works best when all parties are trained in using the same framework. It is essential that healthcare leaders and professionals from across the health disciplines work together to ensure good clinical handover practices are developed and maintained. Organisations, including universities and hospitals, need to invest in the education and training of health professional students and health professionals to ensure good quality handover practice. Using ISBAR as a framework, the purpose of this paper is to highlight key elements of effective clinical handover, and to explore teaching techniques that aim to ensure the framework is embedded in practice effectively.

## Background

Clinical handover is defined as “*The exchange between health professionals of information about a patient accompanying either a transfer of control over, or of responsibility for, the patient”* [1]. It is one of the most critical steps in a patient’s journey [2] and is a core skill that needs to be taught to health professional students and junior clinicians. Performed well, clinical handover should ensure that lapses in continuity of patient care, errors and harm are reduced in the hospital or community setting [2]. The key function of clinical handover is to improve the effectiveness of the actions taken by the recipient/s [1]. Despite its importance, clinical handover is often poorly performed – with potentially serious consequences for the patient [1]. Australian research suggests that critical detail is often omitted during handover, and included information is sometimes irrelevant [3, 4]. Although essential to safe medical practice and provision of excellence in patient care [2, 5, 6], training in clinical handover is often inadequate and not always included in university healthcare curricula [3]. Using ISBAR as a framework, the purpose of this paper is to highlight key elements of effective clinical handover, and to explore teaching techniques that aim to ensure the framework is embedded in practice effectively.

### ISBAR

**Fig. 1:**
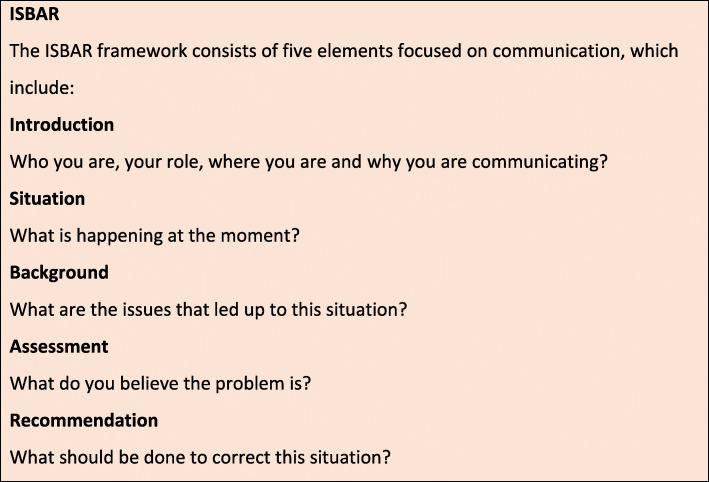
ISBAR framework [9–12]

Evidence suggests the use of structured, standardised frameworks for handover improves information transfer and patient outcomes [7]. In order to improve handover, a number of structured formats have been developed. One example is the I-PASS handover system, developed for use in paediatrics (Illness severity, Patient summary, Action list, Situation awareness and contingency planning, Synthesis by the receiver) [8]. However, one of the most widespread and well-studied frameworks is ‘ISBAR’ (Fig. 1) [9–12]. ISBAR is based on ‘SBAR’ – a system developed by the US Navy to ensure clear, precise communications between nuclear submarines. The ISBAR framework, endorsed by the World Health Organisation provides a standardised approach to communication which can be used in a wide range of clinical contexts, such as shift changeover, patient transfer for a test or an appointment, inter-hospital transfers and escalation of a deteriorating patient [9, 10]. In the hospital setting, ISBAR has been shown to increase transparency and accuracy when practicing interprofessional handovers [10, 12]. ISBAR has also proven to be a successful tool for handover in rural and remote Australian settings [11].

Clinical handover works best when all parties are using the same framework [13] and ISBAR provides a shared model for the transfer of relevant, succinct information between clinicians [13]. By providing a clear and standardised framework, it can assist in reducing the power differences that may hinder the transfer of information [13]. Information transfer may include: doctor to doctor; nurse to nurse; doctor to nurse; allied health to doctor; nurse to allied health. ISBAR can be used in a number of interactions, such as shift change, inter-hospital transfers, reports and briefings, medical emergencies, and patient discharge to community services. This approach doesn’t only apply to verbal communication, but can also be used in written forms, including reports, memos, radiology request forms, and referral documents. The structured framework of ISBAR is used extensively within the Australian healthcare system [12–14].

### Tips for preparing for ISBAR

**Fig. 2:**
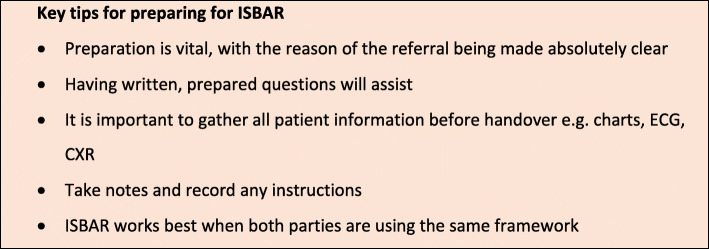
Key tips for preparing for ISBAR [12, 13]

There are important elements to consider in the clinical handover process. Handover must include transfer of accountability for patient care, and the confidentiality of patient information must be maintained. Key tips for preparing for ISBAR are listed in Fig. 2 [12, 13].

**Fig. 3:**
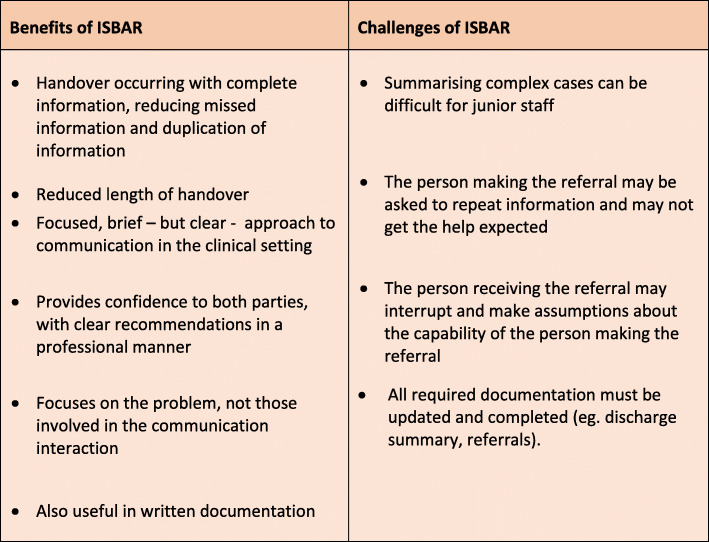
Benefits and challenges of using ISBAR

The benefits and challenges of using ISBAR are listed in Fig. 3 [13]. Challenges can include the complexity of patient cases, and ensuring the person receiving the handover has understood correctly. To help overcome challenges, face to face handover is recommended wherever possible, allowing for interaction and clarification of information [13].

Flow of patient information is vital to patient safety, and a balance between efficiency and comprehensiveness is required [6]. In planning and organising clinical handovers, it is essential to consider:

1. Who should be involved?
2. When should it take place?
3. Where should it take place?
4. How should it occur?
5. What information should be handed over?

**Fig. 4:**
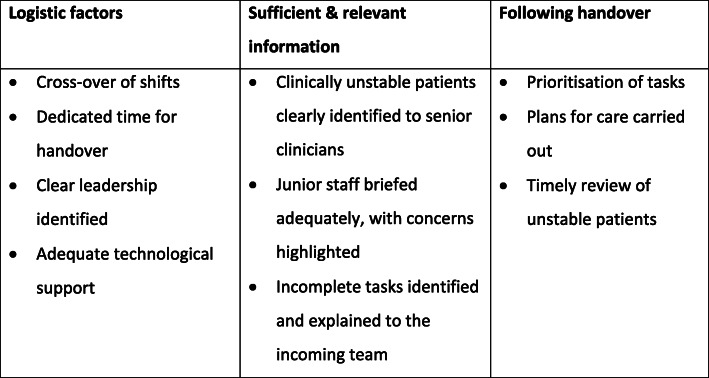
Key elements in helping to ensure continuity of patient information and care during and following clinical handover [6]

Staff rosters should ensure shifts cross over, with dedicated handover time, and clear leadership practices. Sufficient and relevant patient information is required during handover. Junior members of staff must be adequately briefed, and clinically unstable patients must be highlighted to senior clinicians [6, 15]. Any incomplete tasks must be clearly understood by the incoming healthcare team. Similarly, once handover is complete, information must be acted upon. Tasks need to be prioritised; patient care plans need to be acted upon; and unstable patients need to be monitored and reviewed in a timely manner [6]. Key elements in helping to ensure continuity of patient information and care during and following clinical handover are summarised in Fig. 4.

The use of electronic systems may assist with the efficiency of handover. Within the hospital setting, some functions of the electronic handover tools include provision of the name and contact details of covering doctors for each consultant; and identification of patients in need of review, and outstanding tasks [6]. Benefits of using electronic systems include the ability to have multiple users, linkage of information; immediate update of information; and assistance with further planning and prioritisation.

### Education, training and practice in clinical handover

Although ISBAR is proving to be a valuable handover tool, for it to be successful, it must be effectively taught, and health professionals must be adequately trained in its use [10]. A recent systematic review of education interventions [16] revealed that although many handover studies mention interprofessional practice, educational interventions occur predominantly within the uni-professional context. This systematic review also highlighted inadequate reporting of educational interventions and outcomes, thus impeding replication studies [16]. Teaching approaches were mostly based on simulation and role-play, often sequenced with didactic teaching, video examples of handover, discussion and reflection. The systematic review concluded that a greater emphasis on multidisciplinary training of handover would add value to educational interventions [16].

Organisations, including universities and hospitals, need to invest in the education and training of health professional students and health professionals to ensure good quality handover practice. However, due to time constraints in university curricula, and in hospital training, teaching and practice in clinical handover may not be prioritised. By embedding the teaching of handover within the university healthcare curricula, students are able to develop and practice required communication skills to better prepare for their future roles [17, 18]. Along with further training in the workforce, with dedicated teaching time, a well-led handover session itself, provides a useful setting for clinical education [6]. There are a number of online tools and videos available to assist with the teaching of ISBAR. For example:

- The New South Wales Excellence Commission, Clinical Handover [19] http://www.cec.health.nsw.gov.au/improve-quality/clinical-handover
- ISBAR patient safety [20]: https://www.youtube.com/watch?v=h0Ol6CiJAZw
- ISBAR: identifying and solving barriers to effective handover in inter-hospital transfer [21] https://www.youtube.com/watch?v=1Wl9qogPw1E

**Fig. 5:**
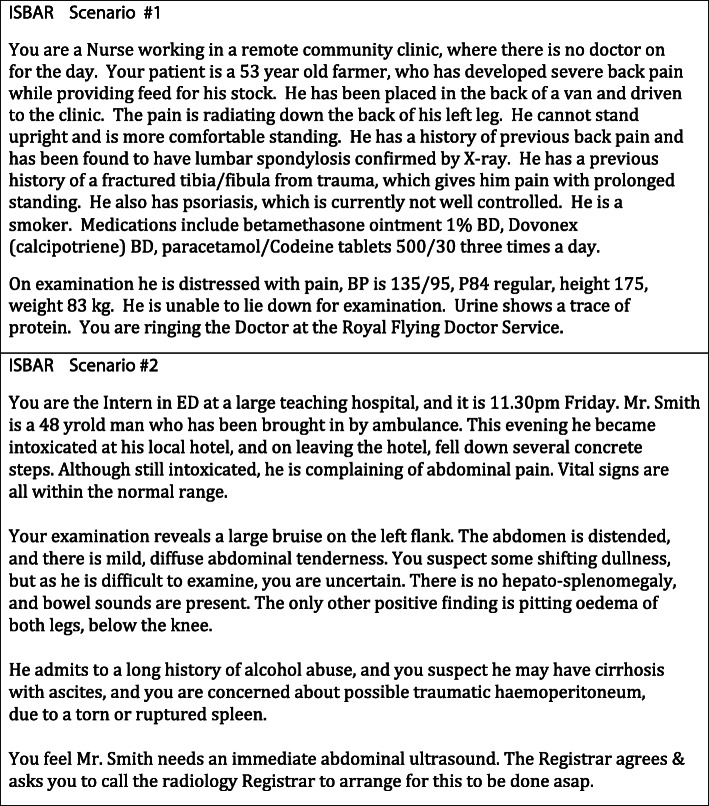
Examples of ISBAR scenarios

Support in education, training, practice, assessment and feedback are essential. Based on our own extensive experience of facilitating clinical handover tutorials, with large interprofessional classes (allied health, nursing, medicine, pharmacy, dentistry), we recommend the following teaching method, which combines large class and small group activities [17, 18]. Students watch suitable ISBAR videos online prior to class, and then attend a face-to-face class, facilitated by a clinical teacher, with interactive discussion. Then in small, interprofessional groups, students use relevant scenarios to participate in simulation/roleplay activities (approximately four students per group), providing an active method of practicing clinical handover. Two examples of scenarios are provided in Fig. 5. Learners can work in pairs to practice giving and receiving a clinical handover. Direct observation, assessment, and feedback, from both peers and an experienced clinician assist in the development of skills [22, 23]. When ISBAR is practiced in larger groups, it is possible for class participants to duplicate the handover, until it is eventually performed to ‘perfection’.

### Feedback and assessment

**Fig. 6:**
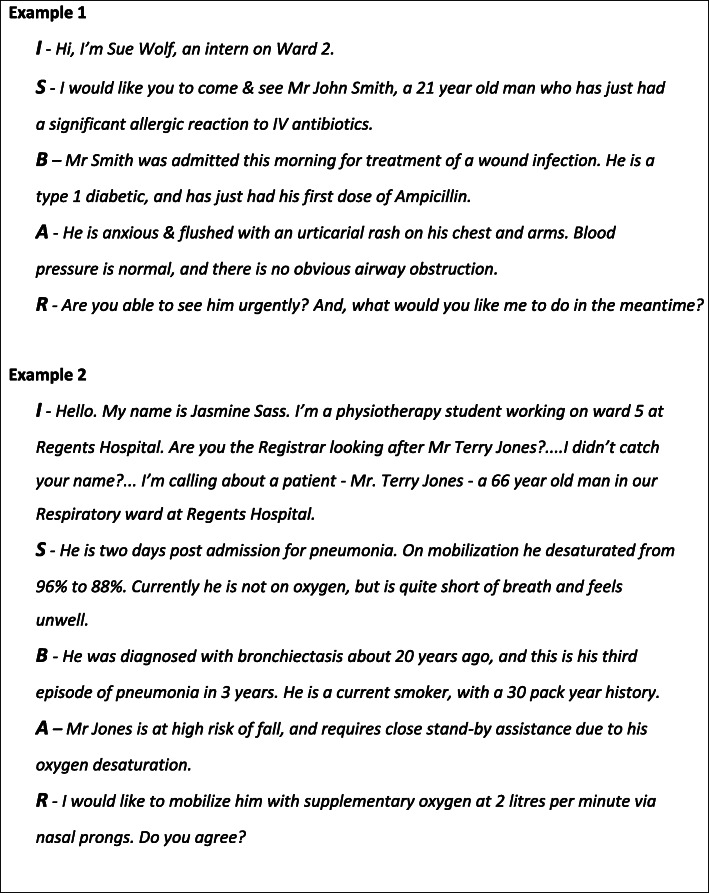
Examples of the use of ISBAR in a role play

Examples of using ISBAR in a roleplay situation are found in Fig. 6. It is important to remember that direct observation of clinical practice, with feedback by an experienced clinician helps to close the gap between current and desired performance by the learner [21, 22]. Additionally, verbal qualitative feedback, with group participation, provides a useful method in teaching clinical handover [17, 18]. For example; “You gave excess information”; “The information was unfocussed”. It is important that the person giving the handover realises that the ‘receiver’ is likely to ask questions, and it is essential to have all available information at hand during ISBAR. Specific assessment tools can also be used. The “Clinical Handover Assessment Tool” (CHAT) was developed by Moore et al. (2017), and is based on the ISBAR handover framework [11]. We have used this model in the assessment of ISBAR performance during small group sessions. The items in this assessment tool are aligned with ISBAR, including, *“Identifies self and position”, “Identifies main problem”, “Gives appropriate history”, “Give appropriate examination/observation”, “Makes logical assessment”, “Makes a clear recommendation*” [11]. A four point scale, ranging from “*Not performed competently*” to *“Able to perform under minimal directio*n” is used to rate the learners’ performance. A global rating is also provided, *“How confident am I that I received an accurate picture of the patient?”*. Importantly, written qualitative feedback is also provided to the learner [11].

### Interprofessional practice and feedback with handover

Peer feedback within the interprofessional context is particularly valuable during interprofessional clinical handover practice activities [17, 18]. Interprofessional activities, within small groups, where participants share experiences within their own field of healthcare offers valuable learning and teaching opportunities, leading to knowledge and skills being socially constructed [17, 18]. Feedback from outside of one’s own health profession can be even more beneficial and meaningful than feedback from within the same discipline. Multidisciplinary feedback on the handover can help to provide an increased understanding of the knowledge, roles and skills of other health professionals; and provide an increased understanding of how this relates to their own health discipline [17, 18]. It also helps participants to reflect on their own technique, terminology and communication methods.

### Importance of ongoing training in clinical handover

The healthcare workforce has seen many changes in recent years, including reduced working hours, and increasing demands for flexibility [24–26]. This is a consequence of better recognition of the impact of doctor fatigue on patient safety, the importance of work/life balance, an ageing healthcare workforce, and the increasing complexity of patient care. In turn, this increases the number of individuals involved in the care of each patient. Therefore the need for concise, accurate clinical handover is imperative [6]. High quality transfer of patient information from team to team is an essential component of good patient care, alongside the expertise of clinicians, teamwork and effective management [27]. However, good practices in clinical handover do not happen by chance. Institutions and organisations (for example, hospitals, universities, training and accreditation bodies) and their leaders, have responsibilities to ensure practice in good clinical handover is achievable [6, 27].

## Conclusion

Effective clinical handover is an essential component of safe patient care to ensure reduction in errors, patient harm, and improve continuity of care. With rapidly changing work patterns within the healthcare workforce, excellence in clinical handover is increasingly important. It is essential that healthcare leaders and professionals from across the health disciplines work together to ensure good clinical handover practices are developed and maintained. Protected teaching time and resources are essential to support staff and students in these endeavours. While a number of tools have been developed to improve handover, we have found the well-researched ISBAR to be an ideal tool to employ for effective clinical handover. However, effective training and practice in the use of ISBAR is essential. Ideally, this training will commence within university healthcare curricula.

### Take-home message

**Table:** 

• ISBAR provides a standardised approach to clinical handover, and can be used in most situations. • For effective handover, think/talk/write and be clear/focused/relevant. • Support for clinical handover training during university and healthcare training is essential to good practice.	

## Data Availability

Not applicable.

## Abbreviations

ISBAR: Introduction, Situation, Background, Assessment, Recommendation
I-PASS: Illness severity, Patient summary, Action list, Situation awareness and contingency planning, Synthesis by the receiver
CHAT: Clinical Handover Assessment Tool
